# Coupled models may help clarify the drivers of observed icesheet melt season shift: comments on Liang *et al.* (2023)

**DOI:** 10.1093/nsr/nwad204

**Published:** 2023-07-26

**Authors:** Xavier Fettweis

**Affiliations:** Department of Geography, University of Liège, Belgium

A new machine-learning-based algorithm deriving melt extent from satellite data [1] has shown that the timing of the melt season over Antarctica has shifted between 1978 and 2020. The later onset and end of the melt season result from complex interactions between the general circulation around Antarctica and sea-ice cover impacting heat exchanges between atmosphere and ocean around the ice sheet. The authors have also demonstrated that this delay in the melt season with respect to maximum solar radiation has decreased the amount of solar radiation absorbed by the snowpack. It has therefore dampened the production of meltwater over recent years. While the authors have clearly identified the drivers of this shift, there are a lot of interactions and positive feedbacks in climatology. By using observations only (satellite or reanalysis as used in [1]), it is impossible to precisely estimate or isolate the role of each climate component in this shift, as changes in one component could trigger or enhance changes in other components.

One solution for performing such an estimate would be to use climate models and, in particular, polar regional climate models coupled with a regional ocean model [2] explicitly simulating the different processes driving the ice-sheet–atmosphere–sea-ice–open-ocean interactions around Antarctica. With this kind of coupled tool, we are able to isolate one process and to estimate its direct and indirect impacts on the other components. By using fixed oceanic conditions (from climatology) in a regional climate model, we could for example estimate the precise role of the recent general circulation changes driven by the Southern Annual Mode (SAM) variability in the timing of the melt season. In the same way, by using atmospheric circulation from a typical year at the lateral boundaries of the regional climate model coupled with an ocean model (forced by the observed ocean at its lateral boundaries), we are able to estimate the impact of the recent sea-ice-extent change on heat exchanges between ocean and atmosphere around the ice sheet. Moreover, if we use a typical annual cycle at the lateral boundaries of the ocean model, we should also be able to estimate the impact of recent Antarctic Ocean warming in these changes. Finally, it will be interesting to check, once both atmospheric and oceanic models are fully coupled, if the coupled model is able to simulate the shift of the melt season observed in [1].

As this melt season shift has recently dampened the production of meltwater, it could therefore have a significant impact on the projected future melt increase. Such changes in the timing of the melt season are currently not suggested in available future projections. It is only longer melt seasons that are projected, in particular over the ice shelves, which will be more affected by the surface melt increase than by the heavier snowfall accumulation due to rising temperatures [3,4]. Therefore, the important role of the atmosphere–ocean interactions highlighted in [1] shows the necessity of having coupled models (atmosphere–ice-sheet–ocean) to better estimate future climate change and how this will impact Antarctica ice-sheet stability. A model resolving one component only, with limited interactions with other ones, could miss important feedback enhancing or mitigating future changes. Such useful coupled models, difficult to implement and expensive in terms of computer time if run at high resolution, are to be developed in the framework of the ongoing European H2020 PolarRES (https://polarres.eu/) and CriceS (https://www.crices-h2020.eu) projects, which aim to study these interactions over Antarctica.

## References

[1] Liang L , Guo H, Liang S et al. Natl Sci Rev 2023; 10: nwad157. 10.1093/nsr/nwad157 PMC10411670 37565193

[2] Huot PV , Kittel C, Fichefet T et al. Clim Dyn 2022; 59: 41–60. 10.1007/s00382-021-06115-x

[3] Kittel C , Amory C, Agosta C et al. The Cryosphere 2021; 15: 1215–36. 10.5194/tc-15-1215-2021

[4] Kittel C , Amory C, Hofer S et al. The Cryosphere 2022; 16: 2655–69. 10.5194/tc-16-2655-2022

